# New Uterine Supporter

**Published:** 1867-10

**Authors:** 


### *New Uterine Supporter.*

Attention is directed to the instrument, invented by Dr. Babcock, whose circular is herewith enclosed. We have as yet, in our private practice, not had occasion to test its merits, but have heard it well spoken of by gentlemen well informed on the subject. Our thanks are due to the inventor for a specimen, which we shall probably soon put to the test of experience.

